# Artificial intelligence as a computational kit for digital biomarker discovery

**DOI:** 10.1515/jtim-2026-0045

**Published:** 2026-06-13

**Authors:** Shenda Hong, Qiang Zhang, Gongzheng Tang, Kuangyi Zhang, Ming Xu

**Affiliations:** National Institute of Health Data Science, Peking University, Beijing, China; Institute of Medical Technology, Peking University Health Science Center, Beijing, China; Institute for Artificial Intelligence, Peking University, Beijing, China; State Key Laboratory of Vascular Homeostasis and Remodeling, NHC Key Laboratory of Cardiovascular Molecular Biology and Regulatory Peptides, Peking University, Beijing, China; Oxford Centre for Clinical Magnetic Resonance Research, Division of Cardiovascular Medicine, Radcliffe Department of Medicine, University of Oxford, Oxford, UK; Big Data Institute, University of Oxford, Oxford, UK; Department of Cardiology and Institute of Vascular Medicine, Peking University Third Hospital, Beijing, China; Beijing Key Laboratory of Cardiovascular Receptors Research, Peking University, Beijing, China; Research Unit of Medical Science Research Management/Basic and Clinical Research of Metabolic Cardiovascular Diseases, Chinese Academy of Medical Sciences, Beijing, China

## Introduction

Digital Biomarkers (DBMs) leverage data captured by digital health technologies—such as wearable devices, smartphones, and medical devices—to objectively measure and interpret physiological and behavioral parameters.^[1]^ These computationally derived indicators span a broad spectrum of concepts, including computational phenotypes, features, embeddings, and “-omics” extensions such as Radiomics,^[2]^ Electrocardiographic Omics (ECGomics)^[3]^, Phenomics,^[4]^ and Multi-omics. Compared with traditional biomarkers obtained biochemically, DBMs represent a significant paradigm shift in healthcare due to noninvasive, continuous, and passive nature.

Recently, artificial intelligence (AI) has emerged as a powerful driver in translational medicine. Its automatic pattern recognition capabilities play a core role in accelerating diagnosis, risk assessment, and personalized treatment. The convergence of DBMs with AI gives rise to a new frontier in digital medicine: AI-derived Digital Biomarkers (AI-DBMs), which harness data-driven intelligence to uncover latent health signatures that are undetectable by conventional methods.

While previous reviews have established the general utility of digital health, this article presents the concept of “Computational Kit” *versus* “Biochemical Kit”. Unlike general data modeling pipelines, this Computational Kit provides a novel modular approach designed to foster a shared language between AI researchers and medical researchers, focusing on the instrumental role of AI in bridging data science and clinical pathways.

## AI-DBMs: A computational kit for uncovering human-invisible biomarkers from high-dimensional medical data

AI-DBMs fundamentally transform the paradigm of health assessment. Traditional biochemical tests are invasive, costly, and provide only a static, single-point snapshot of physiology. In contrast, AI-DBMs (*e.g*., heart rate variability, biological age) can be completely non-invasive and seamlessly embedded into everyday life through ubiquitous digital devices, enabling continuous and real-time dynamic monitoring of health status.

AI-DBMs act as a powerful computational lens by three key factors. (1) Managing Data Overload: AI-DBMs process the vast, intricate streams from evolving wearable sensors that currently exceed human analytical capacity. (2) Expanding the Observational Landscape: AI-DBMs capture multi-dimensional dynamics across temporal (progression), interventional (efficacy), biological (cellular-to-macro), and spatial (population-level) scales. (3) Integrating with Biochemical Biomarkers: AI-DBMs bridge the gap between static laboratory values and continuous, real-world time-series data, providing a more holistic clinical picture.

### Translational potentials and applications

The transformative potential of AI-DBMs lies in their inherent capacity for predictability, quantifiability, and generalizability. For instance, in cardiovascular health, analysis of photoplethysmography (PPG) signals is yielding the AI-derived PPG Age. Specifically, deep learning models process raw PPG signals to achieve high area under the curve (AUC) scores in vascular aging detection. By capturing the subtle, high-frequency morphology of arterial pulses that human observation misses, this AI-DBM provides superior predictive power compared to traditional blood-pressure-based markers.^[5]^ In sleep medicine, AI is capable of annotating continuous sleep depth from polysomnography (PSG) data, revealing novel digital biomarkers for sleep health.^[6]^ In oncology, the field of radiomics utilizes machine learning to extract high-throughput features from medical images, functioning as an AI-DBMs discovery engine that can predict the number of lymph node metastasis in locally advanced gastric cancer.^[7]^ In daily-life health monitoring, video-based vascular analysis collected from smartphones has enabled the identification of a digital biomarker for diabetes.^[8]^ Furthermore, AI has been used to analyze voice signals for the diagnosis and management of respiratory diseases.^[9]^

**Figure 1:**
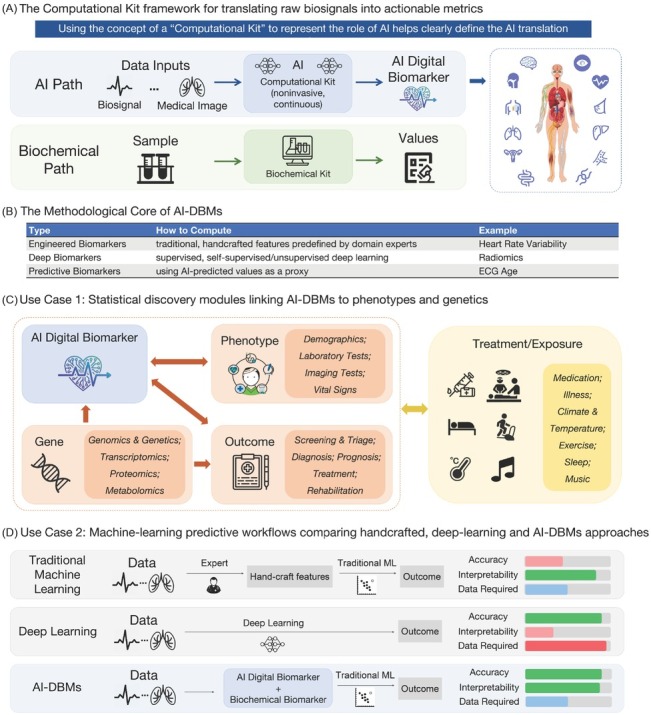
(A) The Computational Kit framework for translating raw biosignals into actionable metrics. (B) The Methodological Core of AI-DBMs. (C) Statistical discovery modules linking DBMs to phenotypes and genetics. (D) Machine-learning predictive workflows comparing handcrafted and deep-learning approaches. AI-DBMs: artificial intelligence-derived Digital Biomarkers; DBMs: Digital Biomarkers. Created using Microsoft PowerPoint.

To further clarify the translation pathway, the role of AI can be conceptualized as a Computational Kit (Figure 1A). For AI researchers, this framework underscores not only the importance of model development, but also considerations such as cost and interpretability. For clinicians, emphasis should move beyond algorithmic innovation to include clinical pathway integration, regulatory-grade validation such as randomized controlled trials (RCTs), and the harmonized use of the computational kit alongside other clinical kits.

### The methodological core of AI-DBMs

The methodological core of AI-derived DBMs lies in leveraging the computational power of AI to transform large-scale, high-dimensional raw medical data into a low-dimensional, quantifiable, and clinically meaningful set of numeric values. The fundamental distinction between AI-DBMs and conventional AI lies in the shift from “algorithms” to “tools”. While standard AI algorithms typically require users to manually collect data and undergo the intensive process of training their own models, AI-DBMs as an AI tool enable researchers to extract biomarkers directly from raw data.

AI-DBMs typically follow three approaches: (1) Engineered Biomarkers. These rely on traditional, handcrafted features predefined by domain experts, such as extracting heart rate variability from Electrocardiogram (ECG) signals. While highly interpretable, these methods can be limited by the constraints of manual feature design. (2) Deep Biomarkers. These utilize deep learning architectures (*e.g*., Convolutional Neural Networks [CNNs], Transformers) to automatically learn and distill powerful and compact representations or embeddings from raw data. These may involve supervised learning strongly linked to outcomes such as disease diagnosis, or self-supervised and unsupervised learning techniques to discover unknown structures, hidden patterns, or clusters within the data. (3) Predictive Biomarkers. These are first trained on a concrete target using supervised learning. The resulting predicted values are then used as a proxy for different downstream tasks. Notably, the concept of the proxy may shift from the original target. For example, using vascular age derived from PPG signals as a broader indicator of cardiovascular health.

The comparison of above three approaches is shown in the Figure 1B. Engineered biomarkers are prioritized for smaller datasets where expert-driven transparency is essential. Deep biomarkers excel with massive data, leveraging deep learning to uncover “human unknown” patterns that manual designs might miss. Predictive biomarkers offer a unique clinical pathway by transforming AI predictions into intuitive proxies for broader health assessment.

Some representative open-source developments for AI-DBMs include PyRadiomics for imaging-based radiomic feature extraction (https://pyradiomics.readthedocs.io) and ECGomics for biosignal-based digital biomarker engineering (https://github.com/PKUDigitalHealth/ECGomics). Rather than being a single software package, AI-DBMs tools includes modular components such as data interfaces, feature extraction modules, and interpretability tools. In this context, existing established frameworks—including radiomics toolkits and data-driven modeling pipelines—are essentially specific technological implementations of Computational Kit concept.

### Use case 1: Statistical discovery modules linking AI-DBMs to phenotypes and genetics

As shown in Figure 1C, starting with medical data such as ECG, AI-DBMs are first extracted using AI algorithms. These digital biomarkers bridge multi-scale biomedical information, linking upward to patient phenotypes (including demographics, lab tests, imaging, and vital signs) and downward to the genetic and molecular characteristics. Most crucially, DBMs connect interventions and exposure factors (*e.g*., medication, season, climate, temperature, exercise, music, illness, and sleep) and clinical outcomes (*e.g*., screening, triage, diagnosis, prognosis, treatment recommendation, and rehabilitation). Through such multidimensional association analyses, AI-DBMs enable mechanistic discovery, comprehensive health assessment, and outcome prediction. AI-DBMs provide a data-driven foundation for personalized treatment and intervention measures.

### Use case 2: Machine-learning predictive workflows comparing handcrafted, deep-learning and AI-DBMs approaches

As shown in Figure 1D, traditional ECG diagnostic models rely on handcrafted features manually extracted by domain experts. While highly interpretable, these methods suffer from limited accuracy constrained by feature design. In contrast, end-to-end deep learning models directly feed raw ECG signals into deep neural networks. While such models achieve high accuracy, they typically lack interpretability due to their “black-box” design and require large datasets for training. The AI-DBMs approach represents a balanced and clinically practical solution. It first leverages AI to extract quantifiable DBMs from the raw data, together with biochemical biomarkers, and then feeds these biomarkers into predictive models. This two-stage method significantly enhances model interpretability by extracting intermediate features while maintaining high accuracy. Furthermore, compared to end-to-end deep learning, it often requires less data, providing a superior pathway for clinical application and translation in real-world digital healthcare environments.

## Current challenges and considerations

However, the transition of AI-DBMs to clinical deployment still faces hurdles. Technical barriers include the reproducibility crisis in machine learning and a lack of cross-device data standardization. Furthermore, moving AI-DBMs into practice requires validation through RCTs and adherence to Food and Drug Administration/ Conformité Européenne (FDA/CE) approval standards. Finally, ethical dimensions such as data privacy and the potential for demographic bias in training sets must be addressed to ensure equitable healthcare delivery.

## Future directions

The future development of AI-DBMs will follow several transformative trends. First, the emergence of foundation models, pre-trained on massive digital health datasets (*e.g*., 10-million ECG records^[10]^, will provide general and robust data representations, fundamentally changing biomarker discovery. Second, multi-modal data integration will merge diverse sources (images, ECG, voice, *etc*.) to construct unified, comprehensive AI-DBMs. This holistic approach will extend beyond single-disease diagnosis, enabling precise risk prediction and personalized medicine. Third, the AI-DBMs approach encourages the use of Explainable AI (XAI) and standardized data interfaces to provide the transparency necessary for clinical trust. For example, providing visual heatmaps that highlight the specific segment of an ECG waveform leading to a high-risk prediction can help clinicians understand the mechanistic basis of the model. Such transparency not only increases trust and enables more informed patient management, but ultimately accelerates regulatory approval and unlocks the full potential for biological discovery.
